# Common Radiographic Findings in Moroccan Working Equids: A Retrospective Study (2015–2022)

**DOI:** 10.3390/vetsci13010060

**Published:** 2026-01-08

**Authors:** Zineb EL Brini, Ichrak Mhar, Fatima Ezzahra Bouktaib, Mohamed Piro, Carola Daniel, Hassan Alyakine

**Affiliations:** 1 Department of Medicine, Surgery, and Reproduction, Agronomy and Veterinary Institute Hassan II, Rabat 10000, Morocco; m.piro@iav.ac.ma (M.P.); h.alyakine@iav.ac.ma (H.A.); 2 The Royal (Dick) School of Veterinary Studies, The Roslin Institute Easter Bush Campus, Midlothian EH25 9RG, UK; v1imhar@ed.ac.uk (I.M.); carola.daniel@ed.ac.uk (C.D.); 3 Laboratory of Biodiversity, Ecology and Genome, Department of Biology, Faculty of Sciences, University Mohammed V, B.P 1014 RP, Rabat 10100, Morocco; fatimaezzahra.boukt@gmail.com

**Keywords:** working equids, radiography, musculoskeletal disorders, foot-related changes, fractures, periosteal new bone formation, animal welfare

## Abstract

Working equids are at high risk of musculoskeletal disorders due to strenuous labor, repetitive tasks, and harsh environmental conditions. This retrospective study describes the distribution of radiographically detected musculoskeletal findings in working equids presented to four Society for the Protection of Animals Abroad (SPANA) centers in Morocco, based on 498 animals and 1125 radiographs collected between 2015 and 2022. The study population was mainly composed of horses (78.1%), followed by donkeys (15.3%) and mules (6.6%). Most were males (65.7%), and the majority were between 5 and 15 years old (60.4%). The distal limb (foot, pastern, and fetlock) was the most frequently examined region (62.7%). Among the animals reviewed, 381 (76.5%) exhibited at least one radiographically detected abnormality, while 117 (23.5%) showed no visible osseous change. The most frequent findings included foot-related changes (36.2%), defined as non-fracture podiatric abnormalities, fractures (29.7%), and periosteal new bone formation (22%). Less frequent findings were degenerative joint disease (8.1%), joint subluxation or luxation (1.6%), epiphyseal abnormalities (1.6%), and angular deformities (0.8%). These results provide an overview of radiographically detected osseous changes in working equids under field conditions. They highlight the diagnostic value of radiography in low-resource environments and provide a basis for future field-based studies.

## Simple Summary

Working equids, including horses, donkeys, and mules, are essential for daily transportation and labor in many Moroccan communities. However, they are often exposed to demanding conditions that predispose them to musculoskeletal injuries, including foot-related changes, fractures, and other skeletal disorders. In this study, we reviewed more than 1100 radiographs from nearly 500 equids examined at four Society for the Protection of Animals Abroad (SPANA) centers in Morocco. Foot-related changes were the most frequent findings, followed by fractures and periosteal new bone formation. Less common conditions included degenerative joint disease, limb deformities, and other bone-related abnormalities. Radiography proved to be a key diagnostic tool for identifying these disorders and supporting clinical decision-making in low-resource settings. These findings provide descriptive information on radiographically detectable musculoskeletal conditions in working equids and offer a basis for future research under field conditions.

## 1. Introduction

Horses, donkeys, and mules are essential to the livelihoods of many communities, particularly in low- and middle-income countries, where they contribute significantly to employment, transportation, agriculture, and household tasks [1,2]. According to the Food and Agriculture Organization [3], the global population of working equids—including donkeys, horses, and mules—is estimated at approximately 112 million, with nearly 39 million residing in low- and middle-income countries (LMICs), supporting the livelihoods of approximately 600 million people worldwide, especially in marginalized and economically disadvantaged areas [4].

In Morocco, the working equid population exceeds one million. As reported by the Directorate of Strategy and Statistics [5], this includes approximately 187,029 horses, 949,604 donkeys, and 324,069 mules. These animals are deeply integrated into the socio-economic structure of both rural and peri-urban areas, where they are primarily used for agricultural work, urban and peri-urban transport, cart pulling, construction-related activities, equestrian tourism, and cultural ceremonies. Their endurance and versatility make them indispensable work partners in daily life.

Due to the physically demanding nature of their work, often performed for long hours under challenging working environments, including hard or uneven working surfaces, working equids are particularly susceptible to musculoskeletal disorders, including chronic joint and soft tissue abnormalities and overuse-related injuries associated with repetitive mechanical loading [6,7,8]. Such health issues can compromise animal welfare and pose direct risks to the livelihoods of dependent communities, reinforcing the relevance of the One Health approach, in line with internationally recognized animal welfare frameworks [2,4].

Radiography plays a key role in evaluating musculoskeletal health in equids, offering a practical and cost-effective means of detecting skeletal abnormalities, assessing injury severity, and informing treatment plans [9]. However, despite its widespread use in clinical settings, there is a notable lack of research describing radiographically detected musculoskeletal findings in working equids, particularly within the socio-economic context of Morocco.

This study aims to identify and categorize radiographically detected musculoskeletal abnormalities in Morocco’s working equids through a retrospective analysis of radiographic data. By quantifying the distribution of these skeletal changes within the examined population, the study explores patterns of abnormalities in relation to working conditions and the socio-economic context. As the analysis is restricted to equids referred for imaging, the findings should be interpreted within this specific population and not extrapolated to the wider working-equid population. Nevertheless, these findings provide a basis to inform targeted health interventions and guide future research and outreach strategies in similar working-animal settings.

## 2. Materials and Methods

The present study aimed to describe the distribution and frequency of radiographically detected musculoskeletal abnormalities observed in working equids referred for imaging examination at four Society for the Protection of Animals Abroad (SPANA) centers in Morocco—Marrakech, Casablanca, Chemaia, and Khemisset—between 2015 and 2022. SPANA is a non-profit organization providing free veterinary care and welfare support for working equids in Morocco.

Only animals presenting clinical signs of lameness or locomotor abnormalities were selected for radiographic evaluation. Lameness was identified based on routine locomotor examination performed by SPANA veterinarians, including visual gait assessment, palpation, and flexion tests. Diagnostic anesthesia (nerve or intra-articular blocks) was not performed systematically under field conditions. The number of radiographs per case varied depending on the anatomical region and on the ability to reach a diagnostic conclusion, in accordance with the standard clinical workflow of SPANA centers. In some cases, multiple anatomical regions were radiographed in the same animal. For descriptive analyses, each radiographic abnormality was recorded and classified independently by lesion category, while animal-level analyses were performed based on the presence or absence of at least one radiographic finding per individual.

Radiographs were obtained using Agfa computed radiography (CR) systems provided by BCF Technology (Bellshill, Scotland, UK). Standard lateromedial and dorsopalmar/dorsoplantar projections were acquired whenever possible, with oblique or skyline views performed when clinically indicated. Exposure parameters were selected according to manufacturer recommendations and adjusted to the animal’s size and the anatomical region examined. Anti-scatter grids were not available in SPANA centers and were therefore not used.

All images were reviewed by two veterinarians experienced in equine orthopedics and diagnostic imaging: the first author, with over seven years of experience, and a senior clinician with approximately thirty years of professional practice in diagnostic imaging. The evaluation was performed blindly, without access to clinical history or examination findings, to minimize interpretation bias. As a consequence, no clinical–radiographic correlation could be performed, and the analysis was therefore limited to the description of radiographic findings. Lesions were identified independently by both readers according to predefined diagnostic criteria proposed by Butler et al. [10].

### 2.1. Inclusion and Exclusion Criteria

To ensure consistency in lesion classification, predefined inclusion and exclusion criteria were applied across all types of radiographic abnormalities. These criteria were intended to standardize the analysis and minimize bias related to image quality or lesion overlap.

Only radiographic abnormalities that were clearly visible and consistent with pathological changes were included, whereas incidental or doubtful findings were excluded from the analysis. The classification of lesions followed the diagnostic principles described by Butler et al. [10], providing a standardized and widely accepted framework for the interpretation of equine radiographic findings. Radiographs of insufficient diagnostic quality (e.g., severe motion artifacts, inadequate exposure, or non-diagnostic projections) were excluded from the analysis.

- Foot-related changes. Radiographs were included when they showed podiatric abnormalities such as ossification of the ungular cartilages, abnormal hoof conformation (e.g., excessively long toe, low or underrun heels), or laminitic changes (increased distance between the dorsal hoof wall and the distal phalanx, dorsal distal phalanx modeling, or rotation). Changes affecting the solar border of the distal phalanx were described as solar margin remodeling, which could include an irregular contour, blunting, or focal areas of bone lysis visible on radiographs, and did not meet the radiographic criteria for laminitis. Fractures involving the foot were excluded from this category and classified separately. Changes in the hoof capsule were described as associated morphological features and were not considered primary osseous lesions.
- Fractures. Defined as visible discontinuity of the cortical bone or fragmentation of osseous structures, regardless of anatomical location or orientation.
- Periosteal new bone formation. All forms of periosteal new bone formation (smooth, irregular, multilamellar, or extensive) were grouped under a single category.
- Degenerative joint disease (DJD). Included radiographic evidence of periarticular osteophytes or enthesophytes, subchondral sclerosis, narrowing or collapse of the joint space, with or without partial ankylosis.
- Subluxation/Luxation. Partial or complete displacement of articular surfaces relative to one another, confirmed on orthogonal projections.
- Epiphyseal conditions. Included developmental abnormalities of the growth plate, such as incomplete ossification, physeal irregularities, and metaphyseal flaring in young animals.
- Angular limb deformities. Defined as varus or valgus deviations in the frontal plane, confirmed radiographically by misalignment of the limb axis and asymmetry of the physis or metaphysis on dorsopalmar/dorsoplantar views.

### 2.2. Statistical Analysis

Statistical analyses were performed using R software (version 4.3.2) [11] within the RStudio environment (version 2024.04.1). Animals without recorded findings were coded as “0”, and those showing at least one radiographic finding were coded as “1”.

Age was standardized and classified into three categories: <5 years, 5–15 years, and >15 years. The frequency of radiographic findings was calculated as the proportion of affected animals, with exact 95% confidence intervals (CI).

Associations between the presence of findings and categorical variables (age, sex, species) were evaluated using Chi-square (χ^2^) tests, with *p* < 0.05 considered statistically significant.

A secondary analysis assessed each finding category separately, comparing distributions across variables. Rare findings were described qualitatively and interpreted with caution.

## 3. Results

### 3.1. Characteristics of the Population Studied

Data were collected from a population of 498 equines, yielding a total of 1125 radiographs. The distribution of equine species within the sample revealed that horses were overrepresented, with 78.1% (n = 389). Donkeys comprised 15.3% (n = 76), while mules represented 6.6% (n = 33) (Table 1).

The study population consisted of 65.7% males (n = 327) and 30.5% females (n = 152). Age distribution showed that 21.7% (n = 108) of the individuals were under 5 years old, 60.4% (n = 301) were between 5 and 15 years, and 15.7% (n = 78) were over 15 years of age, while sex and age data were missing for 3.8% (n = 19) and 2.2% (n = 11) of the population, respectively (see Supplementary Table S1).

### 3.2. Most Common Conditions Determined Radiographically

The following results describe radiographically detected osseous abnormalities. The frequency of radiographed anatomical regions varied notably across the study population (see Supplementary Table S1). The distal limb including the foot, pastern, and fetlock was the most frequently examined region, representing 62.7% (n = 312/498) of cases. Other anatomical regions were less frequently examined, including the metacarpus/ metatarsus (11.4%, n = 57), the carpus (9.6%, n = 48), the radius/ulna (5%, n = 25), and tarsus (4.4%, n = 22). Among the 498 cases reviewed, 23.5% (n = 117) showed no radiographic evidence of osseous abnormalities. In contrast, 76.5% (n = 381) exhibited a total of 416 pathological lesions, documented from the evaluation of 1125 radiographic views.

**Figure 1:**
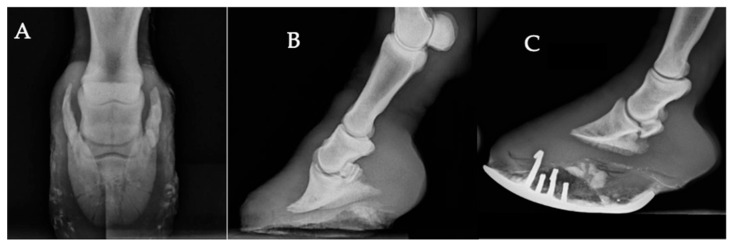
Radiographic views of the equine foot illustrating common radiographic findings. (**A**) Dorsoproximal–palmarodistal oblique view showing marked ossification of the lateral ungular cartilage. (**B**) Lateromedial view showing an increased distance between the dorsal hoof wall and the dorsal surface of the distal phalanx, together with solar margin remodeling and an elongated hoof capsule, radiographic features suggestive of chronic laminitis. A mild subluxation of the distal interphalangeal joint is also present. (**C**) Lateromedial view showing a markedly long toe, excessive sole thickness, and a curved horseshoe conforming to the distorted sole, consistent with long-standing improper trimming.

Foot-related changes were the most frequently observed radiographic changes, with an overall proportion of 36.2% (138/381). The most frequent finding was ossification of the ungular cartilages, observed in 50% of affected animals (69/138) (Figure 1A). Abnormal hoof conformation, characterized by overgrowth and imbalance of the hoof capsule, was present in 39.1% (54/138) (Figure 1C), while solar margin modeling (20.3%, 28/138) and radiographic features suggestive of chronic laminitis (10.1%, 14/138) were also identified (Figure 1B).

**Figure 2:**
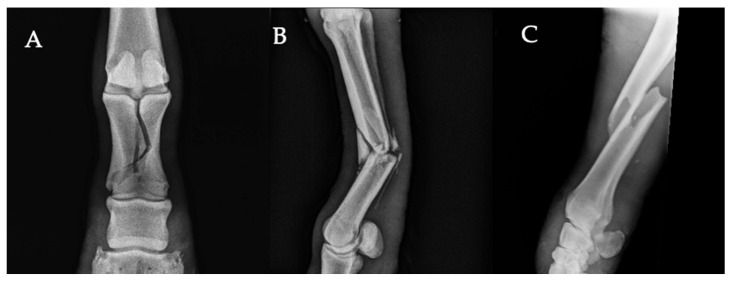
Radiographic views illustrating common fractures in working equids. (**A**) Dorsopalmar radiographic view of the pastern showing a complete, articular, mildly displaced spiral sagittal fracture of the proximal phalanx, with a double radiolucent fracture line extending through both the dorsal and palmar cortices. (**B**) Lateromedial radiograph of the third metacarpal bone showing a complete, bicortical, non-articular fracture of the diaphysis, with displacement between the proximal and distal segments. (**C**) Lateromedial view showing a severely displaced, complete oblique diaphyseal fracture of the radius, with marked overriding between the proximal and distal portions of the bone.

Fractures accounted for a significant proportion of the pathological lesions observed, with a frequency of 29.7% (113/381). The proximal phalanx was the most frequently affected bone, representing 31% (35/113) of fracture cases, followed by fractures of the third metacarpal/metatarsal bone (17.7%, 20/113), the radius (12.4%, 14/113), and the distal phalanx (7.1%, 8/113). Examples of these fracture types are illustrated in Figure 2A–C. Other less frequently affected bones included the tibia, humerus, and ulna. Details of fracture localization and distribution are provided in the Supplementary Table S1.

**Figure 3:**
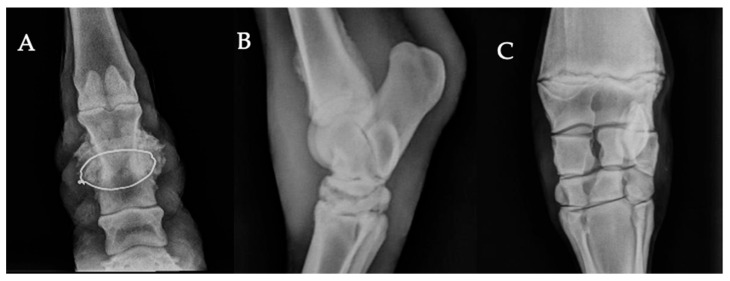
Periosteal and joint-related radiographic findings observed in working equids. (**A**) Dorsopalmar radiograph of the pastern showing extensive, irregular periosteal new bone formation surrounding a radiopaque foreign body (wire) embedded at the mid-portion of the proximal phalanx (P1). The exuberant periosteal response is compatible with chronic reactive bone formation secondary to soft-tissue trauma. (**B**) Lateromedial radiograph of the tarsus demonstrating significant narrowing of the distal intertarsal joint space with associated subchondral osteolysis, and reduced joint space of the tarsometatarsal joint, suggestive of advanced degenerative joint disease (DJD; bone spavin). Periosteal new bone formation is evident along the cranial and caudal cortices of the distal tibia. (**C**) Dorsopalmar view of the distal radius and carpus showing widening of the distal radial physis and mild medial and lateral metaphyseal flaring; the central axes of the radius and metacarpus are not aligned, consistent with angular limb deformity.

Periosteal new bone formation was identified in 22.0% (84/381) of affected animals (Figure 3A–C). Most lesions were located in the pastern region (59.5%, n = 50) (Figure 3A), followed by those observed along the cortical surface of the third metacarpal or metatarsal bone (23.8%, n = 20). Degenerative and developmental joint disorders were also observed among the study population. Degenerative joint disease (DJD) accounted for 8.1% (31/381) of affected animals (Figure 3B). The joints most frequently involved were the fetlock and the carpus, each representing 35.5% (11/31) of DJD cases, followed by the tarsus, accounting for 22.6% (7/31). Joint luxation or subluxation (Figure 1B) and epiphysitis (Figure 3C) were less common, each representing 1.6% (6/381) of cases. Angular limb deformities, characterized by visible deviation of the limb axis, were the least frequent findings, occurring in 0.8% (3/381) of the affected population.

### 3.3. Statistical Analysis Results

Analysis of the distribution of radiographic findings across demographic variables revealed no statistically significant association between the overall occurrence of radiographic abnormalities and either age (χ^2^ = 2.49; *p* = 0.288) or sex (χ^2^ = 1.29; *p* = 0.526), whereas species showed a significant difference (χ^2^ = 9.62; *p* = 0.008). The proportion of animals showing at least one radiographic finding remained high across all age groups (68.5–74.4%) and genders (65.1% in females, 68.5% in males), with the highest values observed in mules (87.9%), followed by donkeys (73.7%) and horses (64.0%) (Table 1).

When examining individual radiographic categories, age significantly influenced the distribution of findings (χ^2^ = 60.6; *p* < 0.001), while sex remained non-significant (χ^2^ = 11.1; *p* = 0.68). Younger animals (<5 years) most frequently presented fractures (30.6%) and foot-related changes (19.4%), whereas periosteal new bone formation (14.8%) and epiphyseal conditions (5.6%) were less common. Animals aged 5–15 years predominantly showed foot-related findings (30.6%) and fractures (18.9%), with periosteal new bone formation (16.0%) also observed. Older animals (>15 years) exhibited a higher proportion of fractures (28.2%), periosteal new bone formation (24.4%), and foot-related changes (26.9%) (Table 2).

No significant variation in the type of radiographic findings was observed between sexes. Foot-related changes (32.9%) and fractures (21.7%) were similarly distributed between females and males (25.4% and 23.6, respectively), while %, other categories remained low and comparable (Table 2).

Species comparisons showed significant differences in the distribution of radiographic findings (χ^2^ = 34.7; *p* = 0.002): mules exhibited higher proportions of fractures (42.4%) and periosteal new bone formation (27.3%), donkeys showed more periosteal new bone formation (17.1%) and luxation/subluxation (3.9%), while horses had the highest proportions of foot-related changes (29.3%) and new bone formation (15.9%) (Table 2).

Overall, species emerged as the main factor influencing the distribution of skeletal radiographic findings among the examined animals.

## 4. Discussion

### 4.1. Demographic Patterns in Radiographed Animals

Our study reports quantitative differences in species, sex, and age distribution among working equids referred for radiographic examination at various SPANA centers, reflecting animals exposed to sustained physical workloads and work-related mechanical stress. Horses represented the largest proportion of the study population, followed by donkeys and then mules, a disparity likely influenced by multiple factors. In Morocco, horses are often perceived as having a higher economic and functional value compared to donkeys and mules, which could partly influence owners’ decisions to seek veterinary care. Conversely, although studies have shown that donkeys are more prone to lameness [7,12,13], their innate stoicism and high pain tolerance [14,15], often result in subtle or absent clinical signs, making lameness more difficult for owners to detect [16]. Mules represented the smallest proportion of animals presented for radiographic examination in this Moroccan study population. This finding is consistent with international NGO-based data reporting that mules constitute the lowest proportion of equids presented to veterinary services in several low- and middle-income countries [17]. Handling difficulties, combined with safety concerns for both personnel and equipment further limit the feasibility of radiographic examination in donkeys and mules by limiting the number of views obtained or discouraging radiography altogether. Previous studies have reported stress-related negative behavioral responses in working donkeys and mules, with aggressive and avoidance behaviors being more frequently observed in mules under challenging handling and working conditions [18,19]. Consequently, the lower proportion of donkeys and mules undergoing radiographic examination likely reflects a combination of economic, handling-related, and diagnostic constraints.

Males were overrepresented in the study population, consistent with previous findings from Morocco and other countries. Bakkar et al. [20] reported that more than 60% of the 900 working equids examined at the American Fondouk in Morocco were male, a pattern similarly observed by Norris et al. [13] in 2448 equids. This predominance likely reflects traditional management practices, where females are mainly retained for reproduction to maintain herd sustainability, while males are preferred for draft and transport work. Males are generally perceived as stronger and more resilient, leading to their selection for labor-intensive activities that increase the risk of fatigue, injuries, and poorer welfare outcomes. Comparable findings were reported by Assefa et al. [21] in Ethiopia, where 98% of working donkeys were male, underscoring a widespread preference for males based on their greater pulling capacity and economic value.

The age distribution of radiographed cases showed notable variation. Most animals were between 5 and 15 years old, which corresponds to the period considered optimal for active work in equids [22]. At this age, sustained workloads and cumulative mechanical stress increase the likelihood of locomotor problems. A substantial number of animals were younger than 5 years, despite recommendations to avoid work before skeletal maturity [23]. Similar observations have been made in Egypt, where young working equids frequently presented with lameness or bone injuries [24]. Starting work too early and being exposed to excessive strain before full bone development may predispose these animals to long-term orthopedic issues and a reduced working lifespan [25]. In contrast, studies in performance horses indicate that early, controlled exercise can promote adaptive skeletal responses, particularly in bone, and may reduce injury risk when training is progressive and well managed [26,27]. These conditions, however, differ markedly from those experienced by working equids, where workloads are often excessive, poorly regulated, and associated with limited recovery. Conversely, older equids were underrepresented, suggesting that few remain in service long enough to reach advanced age. The degenerative changes observed in this group are consistent with age-related joint disease described previously [28,29,30,31].

Together, these demographic patterns (species, sex, and age) influence the likelihood of presenting musculoskeletal disorders identified through radiographic examination. Since radiographs are performed mainly for lameness investigation, the overrepresentation of certain groups likely reflects differential exposure and owner perception rather than true population prevalence.

### 4.2. Lesions Diagnosed by Radiography

Radiography provided valuable insight into skeletal conditions in working equids but also revealed its diagnostic limits. In many animals, no bone abnormalities were visible, showing that radiography alone cannot fully reflect the clinical status. A thorough and standardized clinical lameness assessment prior to diagnostic imaging is essential to accurately localize the region of pain and optimize interpretation of radiographic findings, particularly under field conditions. Similar findings were described in Ethiopia, where lameness in working donkeys was often linked to muscular or ligamentous problems associated with insufficient rest [21]. These observations highlight the need for a multimodal diagnostic approach that combines radiography with ultrasonographic evaluation whenever possible.

The predominance of hoof and distal limb disorders in radiographed animals underscores the central importance of hoof health in equine welfare and productivity. Numerous field studies from Africa and Asia have consistently identified hoof disorders as major contributors to reduced working capacity and economic loss in equids [6,32,33,34,35,36]. Limited farriery, poor hoof balance, and chronic strain contribute to hoof problems, highlighting the need for regular trimming and owner education. Ossification of the ungular cartilages (OUC), or sidebone, was a frequent radiographic finding in working equids. Marked or irregular ossification may reflect adaptive modeling in response to chronic mechanical stress [37]. Earlier reports noted high prevalence in heavy breeds, but these data came from very different management contexts [38,39]. More recent studies in working donkeys described early OUC, often bilateral and occasionally linked to lameness or complications such as septic pedal osteitis [40].

Ossification of the ungular cartilages (OUC) is considered multifactorial, influenced by limb conformation, hereditary predisposition, hoof imbalance, inadequate farriery, and repetitive concussion on hard surfaces [41,42,43,44,45]. Other factors such as age, sex, body size, workload, and environmental conditions may also contribute [46,47,48], acting together to promote cartilage mineralization visible radiographically.

Laminitis was rarely identified in our radiographic dataset. This contrasts with the high prevalence reported in Moroccan Fantasia horses with chronic bilateral laminitis, largely linked to high-carbohydrate diets and metabolic predisposition [49]. In donkeys, laminitis is more often associated with obesity, sedentary conditions, and overfeeding [50]. These factors are uncommon in working equids, which are usually lean and subjected to sustained physical effort with limited nutrition. This likely contributes to the low occurrence observed here. At SPANA centers, radiography is used as a complementary diagnostic tool in cases of lameness when clinical examination alone does not allow clear characterization of the underlying osseous involvement. Laminitis is usually diagnosed clinically, while radiographs help assess the position of the distal phalanx, foot conformation, and disease progression [51]. When foot radiographs were performed for suspected laminitis or other foot disorders, occasional cases showed solar margin modeling consistent with chronic laminitis or with imbalance of the foot [10].

Fractures were among the most common osseous lesions identified, mainly affecting the phalanges, metacarpal/metatarsal bones, and the radius. Similar studies in comparable working equid populations also reported frequent distal limb involvement [52]. In performance horses, such fractures are often linked to repetitive loading, microdamage, or sudden trauma [53,54,55,56], and similar biomechanical principles likely apply to working equids. Additional factors such as heavy workloads, uneven terrain, poor hoof balance, and inadequate farriery may further increase risk. Trauma from kicks, especially in crowded or poorly managed housing, is also a plausible contributor [57,58].

Periosteal new bone formation was one of the most frequent osseous findings in our study. In horses, these reactions have been reported in association with high-speed exercise [59,60,61], but in working equids they more likely reflect cumulative mechanical strain and localized trauma rather than sport-related loading. The pastern region was the most commonly affected site, where such lesions are typically referred to as ringbone [10]. In this region, periosteal reactions may arise from trauma-induced periostitis, enthesophyte formation at tendon or ligament insertions, or chronic remodeling linked to repetitive mechanical stress [10]. In Morocco, external trauma also appears relevant. The use of hobbles made from inappropriate materials, such as wire or baling twine, has been reported as a cause of localized pastern injuries and subsequent periosteal new bone formation [62,63]. Overall, periosteal new bone formation in working equids likely reflects chronic mechanical overload and localized trauma.

The main limitations of this study include its retrospective design and the absence of detailed clinical histories and follow-up data, which limited assessment of lesion progression over time. In addition, the blind radiographic reading without access to clinical history prevented any clinical–radiographic correlation, and the results therefore describe radiographic findings rather than the causes of lameness. Radiographs were obtained only in animals referred for imaging, which may have introduced a selection bias. Variability in the number and quality of radiographs may have influenced the level of detail available for lesion characterization. Despite these limitations, this study highlights the value of radiography under field conditions and provides a robust basis for future prospective investigations.

## 5. Conclusions

This retrospective study provides an overview of the main musculoskeletal disorders identified by radiography in working equids presented to four SPANA centers in Morocco. Foot pathologies were the most frequent findings, highlighting the central importance of hoof health in maintaining mobility, welfare, and work capacity. Fractures and periosteal new bone formation were also common and reflected the demanding working conditions typically described for equids in similar environments.

Radiography proved valuable for objectively documenting musculoskeletal lesions and supporting clinical assessment under field conditions. Promoting regular hoof care, improved farriery, and safer handling practices remains essential to reduce the burden of these conditions.

Future field-based studies integrating clinical, environmental, and management information will help refine these findings and support the development of preventive strategies adapted to low-resource settings.

## Figures and Tables

**Table 1:** Incidence of radiographic abnormalities according to age groups, sex, and species.

Variable	Variables	Number of Cases	Affected Cases	Incidence (%)	CI 95%-Lower	CI 95%-Upper	χ^2^/*p*-Value
Age (years)	<5	108	74	68.5	58.9	77.1	2.49/0.288
	5–15	301	196	65.1	59.4	70.5	
	>15	78	58	74.4	63.2	83.6	
	UN	11	6	54.5	23.4	83.3	
Gender	Female	152	99	65.1	57.0	72.7	1.29/0.526
	Male	327	224	68.5	63.2	73.5	
	UN	19	11	57.9	33.5	79.7	
Species	Donkey	76	56	73.7	62.3	83.1	9.62/0.008 *
	Horse	389	249	64.0	59.0	68.8	
	Mule	33	29	87.9	71.8	96.6	

Incidence (%) refers to the proportion of affected individuals within each age group, sex, and species category. The 95% confidence intervals (CI) provide an estimate of the precision of the incidence rates. Statistical differences between groups were evaluated using the Chi-square (χ^2^) test. *p*-values are presented alongside χ^2^ statistics; values less than 0.05 (denoted by *) indicate statistical significance. UN = Unknown.

**Table 2:** Risk Analysis of Specific Pathologies in Equids According to Age, Sex, and Species.

Risk Factor	Level of Risk Factor	No Examined	A	B	C	D	E	F	G	χ^2^/*p*-Value
Age (years)	<5	108	19.4	30.6	14.8	1.85	1.85	5.56	1.85	60.6/<0.001 *
	5–15	301	30.6	18.9	16.0	7.31	0	0	0.33	
	>15	78	26.9	28.2	24.4	7.69	5.13	0	0	
	NA	11	36.4	9.09	9.09	9.09	0	0	0	
Gender	Female	152	32.9	21.7	11.8	5.26	0.66	0.66	0	11.1/0.68
	Male	327	25.4	23.6	18.6	6.73	1.53	1.53	0.92	
	NA	19	26.3	15.8	26.3	5.26	0	0	0	
Species	Donkey	76	25	29.0	17.1	9.21	3.95	2.63	1.32	34.7/0.002 *
	Horse	389	29.3	19.8	15.9	5.4	0.77	0.51	0.51	
	Mule	33	15.2	42.4	27.3	9.09	0	6.06	0	

Pathologies are coded as follows: A—Foot disorders, B—Fractures, C—Periosteal new bone formation, D—Degenerative joint disease, E—Sub/luxation, F—Epiphyseal conditions, G—Angular deformities. Statistical analysis was performed using the Chi-square test (X^2^). *p*-values < 0.05 (denoted by *) were considered statistically significant. NA = Not Available.

## Data Availability

The original contributions presented in this study are included in the article/Supplementary Material. Further inquiries can be directed to the corresponding author.

## Supplementary Materials

The following supporting information can be downloaded at: https://www.mdpi.com/article/10.3390/vetsci13010060/s1, Table S1: Distribution of radiographic cases by species, age, sex, and anatomical region.
